# Thirty-Day Postoperative Outcomes Following Sugammadex Use in Colorectal Surgery Patients: Retrospective Study

**DOI:** 10.3390/jcm8010097

**Published:** 2019-01-16

**Authors:** Yun Jeong Chae, Han Bum Joe, Juyeon Oh, Eunyoung Lee, In Kyong Yi

**Affiliations:** 1 Department of Anesthesiology and Pain Medicine, Ajou University School of Medicine, Suwon 16499, Korea; yjchae06@hotmail.com (Y.J.C.); joehanbum@naver.com (H.B.J.); itisamy@hanmail.net (J.O.); 2 Department of Biomedical Informatics, Ajou University School of Medicine, Suwon 16499, Korea; eylee@aumc.ac.kr

**Keywords:** acetylcholinesterase inhibitor, delayed discharge, postoperative outcomes, sugammadex

## Abstract

Purpose: Sugammadex rapidly reverses muscle relaxation compared to acetylcholinesterase inhibitors. The long-term outcomes of sugammadex, however, are not well known. We compared 30-day postoperative outcomes following sugammadex and acetylcholinesterase inhibitor use in colorectal surgery patients. Patients and methods: Colorectal surgical patients older than 21 were included in this retrospective study, and were dichotomized according to use of reversal agents, sugammadex (group S), and acetylcholinesterase inhibitor (group A). We assessed 30-day postoperative outcomes, including total length of hospital stay, length of postoperative hospital stay, readmission rate, and delayed discharge rate. Additional parameters included postanesthetic care unit stay time, time to first successful oral intake, unforeseen intensive care unit (ICU) admission rate, postoperative pulmonary complications, and mortality. Results: Among a total of 585 patients, 157 patients remained in each group after propensity score matching. Total length of hospital stay, length of postoperative hospital stay, and readmission rates did not differ between the two groups, while the incidence of delayed discharge was significantly lower in group S (23 (15%) vs. 40 (25%), *p* = 0.017). Other outcomes did not differ between the two groups. Conclusion: We found no difference in 30-day postoperative outcomes following sugammadex and acetylcholinesterase inhibitor use. The only difference between these treatments was the associated incidence of delayed discharge, which was lower in group S.

## 1. Introduction

Residual neuromuscular blockade following surgery increases the incidence of adverse respiratory events, such as hypoxia and reintubation, in the postanesthetic care unit (PACU) and may delay patient discharge [1,2]. The incidence of postoperative residual neuromuscular blockade among surgical patients is significant, with previous studies reporting 22 to 88% [3,4,5]. During abdominal surgery, a deeper neuromuscular block is required to obtain optimal surgical conditions [6]. If the level of blockade is inadequate, muscle tension in the patient’s abdominal wall and diaphragm may result in poor surgical conditions [7]. Deep neuromuscular blockade is also frequently necessary during abdominal skin closure, which can lead to the incomplete reversal of postoperative neuromuscular blocking effects and increased rates of postoperative complications [1]. The complete induction and reversal of neuromuscular blockade is thus vital to both surgical and postoperative success.

Classic reversal agents of neuromuscular blockade are acetylcholinesterase inhibitors, such as pyridostigmine and neostigmine, which are competitive antagonists of the enzyme cholinesterase. Sugammadex, which forms a tight complex with unbound rocuronium, thereby preventing further binding at the neuromuscular junction, acts via a different mechanism than do acetylcholinesterase inhibitors. Even in the context of a deep neuromuscular block, sugammadex achieves rapid, complete, and reliable reversal. Putz et al. [8] compared deep neuromuscular block reversal with sugammadex, and shallow neuromuscular block reversal with an acetylcholinesterase inhibitor, and found faster and more reliable reversal rates with sugammadex in abdominal surgery patients [8]. This suggests that sugammadex provides faster recovery than acetylcholinesterase inhibitors, regardless of rocuronium dosage or level of neuromuscular block. Furthermore, studies have shown that sugammadex results in no residual neuromuscular blockade and faster discharge rates from the PACU when compared to acetylcholinesterase inhibitors [9,10,11,12].

In particular, studies have focused on the effect of residual neuromuscular blockade in the immediate postoperative period and especially during patients’ stay in the PACU [9,10,11,12]. Thevathasan et al. [13], however, reported on the long-term outcomes of residual neuromuscular blockade. These authors found that intraoperative administration of a neuromuscular blocking agent was dose-dependently associated with an elevated risk of 30-day readmission and prolonged hospital costs, due in part to residual neuromuscular blockade effects [13]. Based on these results and those discussed above, we hypothesized that sugammadex would improve long-term outcomes, including 30-day readmission rates, by reducing complications associated with residual neuromuscular blockade. The present study compared 30-day postoperative outcomes, including total length of hospital stay, length of postoperative hospital stay, 30-day readmission rates, and delayed discharge rates, between colorectal surgery cases requiring at least moderate neuromuscular block cases in which sugammadex or acetylcholinesterase inhibitors were used.

## 2. Materials and Methods

### 2.1. Data Sources and Study Population

This study was performed at a single center at the Ajou University Hospital in Suwon, Korea. This study was approved by the Ajou Institutional Review Board on 22 March 2018, and the requirement for written informed consent was waived by the committee (AJIRB-MED-MDB-18-036). The trial was registered at ClinicalTrials.gov (NCT03619759). This retrospective study included colorectal cancer patients older than 21 who have undergone surgery between January 2012 and December 2017. Surgery was performed by a single colorectal surgeon with 30 years of experience. By the surgeon’s request, beginning in May 2015, sugammadex was used for blockade reversal in every colorectal cancer patient who underwent colorectal surgery. No other significant postoperative care changes were noted during the study period.

Patients were divided into sugammadex and acetylcholinesterase inhibitor groups on the basis of their blockade removal treatment. The sugammadex group (group S) included patients who were admitted between January 2016 and December 2017, while the acetylcholinesterase group (group A) included patients admitted between January 2012 and December 2013. Patients who were admitted between 2014 and 2015 were excluded, as sugammadex was used only in selected patients during this time period. Potential selection bias arising from the cost of sugammadex was avoided as all patients treated between January 2016 and December 2017 received the drug. We excluded bedridden patients due to their high rate of neuromuscular disease, as well as patients with reduced renal or hepatic function. Patients who underwent combined operations or emergency operations were also excluded.

Patients were grouped according to the reversal agent used in their case; group S was compared to group A. In group S, sugammadex was used in fixed dose of 1 vial, 200 mg, with no routine use of neuromuscular monitoring. According to our center’s protocol, all patients undergoing general anesthesia with rocuronium received a reversal agent (i.e., acetylcholinesterase inhibitor or sugammadex), and timing of the administration of the reversal agent was based on clinical judgement regarding parameters: Spontaneous breathing, extremity movement, and bucking [14]. This protocol has not changed from 2012 to 2017. Baseline characteristics were collected, included age, sex, body mass index, American Society of Anesthesiologists physical status, comorbidities, operation type (e.g., open or laparoscopic), and anesthesia time. Rocuronium induction dose and infusion doses followed our hospital’s protocol of 0.6 mg/kg and 0.5–0.7 mg/kg/h respectively. While there is no record of the exact dose administered in each case, we assumed that the total dose per kg was proportional to administration time. 

Primary outcomes in the postoperative period included total length of hospital stay, length of postoperative hospital stay, delayed discharge rate, and readmission rate, including emergency room visits, within 30 days post-discharge. “Delayed discharge” was defined as a postoperative hospital stay of more than 11 days. Additional postoperative outcomes included PACU stay time, unforeseen intensive care unit admission rate, time to first successful oral intake (hours), and mortality. Postoperative pulmonary complications were also recorded, and electronic medical record data were collected including discharge summaries, consultation records, and chest X-ray readings.

### 2.2. Statistical Analyses

The baseline characteristics of patients were analyzed using chi-squared tests for categorical variables and unpaired Student’s *t*-tests for continuous variables. Data are presented as mean ± standard deviation for continuous variables, and as numbers and percentages for categorical variables for those patients with complete data. Propensity scores were derived using separate logistic regression models, including age, sex, anesthesia time, comorbidities, and operation type. Propensity scores were then matched to obtain matched patient pairs. We calculated standardized mean differences across groups to assess any remaining imbalance in the matched samples. After 1:1 propensity score matching, statistical analyses were conducted using unpaired Student’s *t*-tests for continuous variables and a chi-squared test for categorical variables; Fisher’s exact tests were applied as needed. All statistical analyses were 2-sided and were performed with SAS statistical software, version 9.4 (SAS Institute, Cary, NC, USA). A *p*-value of less than 0.05 was considered to be statistically significant.

## 3. Results

### 3.1. Baseline Characteristics

A total of 659 patients were screened for study participation. Among them, 74 were excluded due to a history of combined operations (*n* = 53), reduced renal function (*n* = 14), or reduced hepatic function (*n* = 7). Consequently, a total of 585 patients were enrolled in the present study. A total of 201 patients used sugammadex (group S) while 384 patients used pyridostigmine (group A). Demographics before propensity matching were as follows. Mean age was 63.7 ± 11.3 years and 61.5 ± 12.1 years for groups S and A, respectively (*p* = 0.037). The incidence of laparoscopic surgery was higher in group S (19%) than group A (12%) (*p* = 0.012). Total time under anesthesia for group S was 181.0 ± 48.6 min, which was significantly longer than group A, whose total time under anesthesia was 165.1 ± 41.7 min (*p* < 0.001). All other parameters did not differ significantly between the two groups. After propensity score matching of these baseline characteristics, 157 patients remained in each group (Table 1).

### 3.2. Postoperative Outcome Analysis after Propensity Matching

After propensity matching, the total length of hospital stay, length of postoperative hospital stay, and readmission rates did not differ between the two groups, while incidence of delayed discharge was significantly lower in group S (23 (15%) vs. 40 (25%), *p* = 0.017). All other outcomes, including PACU stay time, unforeseen ICU admission rate, mortality, and time to first successful oral intake, did not differ between the two groups. The incidence of pulmonary complications was not significantly different among groups (4 (3%) vs. 1 (1%), *p* = 0.18) (Table 2).

### 3.3. Analysis of Delayed Discharge Cases

Ileus was the most common cause of delayed discharge in the 63 cases included in the present study (45 (71.4%)). The causes of delayed discharge differed between the two groups, with ileus being the primary reason in 90.0% and 39.1% of patients in groups A and S, respectively (Table 3). Other reasons for delayed discharge included wound problems, urinary tract infections, pneumonia, and postoperative bleeding.

## 4. Discussion

The current study revealed that 30-day postoperative outcomes, including total length of hospital stay, length of postoperative hospital stay, and readmission rates, did not significantly differ between patients who received sugammadex or acetylcholinesterase inhibitors for neuromuscular blockade reversal. Furthermore, PACU stay time, unforeseen ICU admission rate, postoperative pulmonary complication, and mortality rates did not significantly differ between the two groups. The sugammadex group, however, exhibited decreased delayed discharge rates.

Sugammadex is well known for its use in reducing the risk of residual neuromuscular blockade [9,15,16,17,18]. This effect often translates to clinically favorable results during postoperative recovery. A number of prior studies have demonstrated that sugammadex induces rapid reversal of neuromuscular blockade, resulting in more rapid extubation and operating room discharge [8,12,16,19,20,21,22]. The postoperative effects of sugammadex following patient discharge from the operating room, however, have been mixed, with some studies reporting faster PACU discharge times [8,10,11], and others reporting no difference [8,17,23,24]. PACU discharge time may be affected by several factors such as bed availability in the ward, physician release, nurses’ decisions, additional test like radiographs, and availability of transport team [25]. Therefore, PACU discharge time may not have entirely reflected the clinical effects of sugammadex. 

Despite some focus on operative and post-operative outcomes, clinical studies assessing patient outcomes following PACU discharge are scarce. Using a retrospective study design, Ledowski et al. reported that overall hospital stays did not differ between patients treated with sugammadex or acetylcholinesterase inhibitors [24]. These results, however, are derived from patients who underwent surgical procedures including orthopedic, plastic, general, and others.

One strength of the present study is the homogeneity of patients analyzed, which include only colorectal patients who required moderate or deep level of neuromuscular blockade and who were surgically treated by a single surgeon. We speculate that a complete reversal of residual neuromuscular blockade by sugammadex might affect postoperative recovery process due to its effects on generalized muscle weakness [13], which lead to differences in long-term patient morbidity. However, the use of sugammadex in the present study did not result in differences in the total length of hospital stay, length of postoperative hospital stay, or readmission rates in a 30-day period. This may be because the long-term effects of sugammadex on postoperative recovery process are smaller than expected to be statistically captured.

Residual neuromuscular blockade may contribute to the development of postoperative respiratory complications [15,26]. Furthermore, complications such as desaturation, upper airway obstruction, and reintubation in the PACU were reduced with use of sugammadex [12,15,24]. The present study’s results, however, show that postoperative pulmonary complications were not significantly different between the two groups, suggesting that there may be no clear advantage in terms of respiratory functional recovery over acetylcholinesterase inhibitor use. Our results also agree with a recently published, large prospective study wherein reversal with sugammadex did not reduce postoperative pulmonary complications [27].

Currently, data on the benefits of sugammadex for the reduction of clinically-relevant morbidity due to residual neuromuscular blockade are limited [17,24,28]. While sugammadex successfully reduces residual neuromuscular blockade, there is a lack of evidence that this effect translates to improvements in long-term outcomes. Several factors may have affected the similar long-term respiratory outcomes in sugammadex and acetylcholinesterase inhibitor groups in the present study. One possibility is that a residual neuromuscular block remained, even following sugammadex use. We did not implement neuromuscular monitoring, and we used fixed dosages of sugammadex (200 mg per patient) when clinical signs were noted. Kotake et al. [14] reported that sugammadex may also induce roughly 3~4% residual paralysis in a clinical setting when no neuromuscular monitoring is used routinely. In a previous, large prospective study, however, neuromuscular monitoring did not result in improved postoperative pulmonary outcomes [27].

One possibility for these mixed results may be the short-lived effects of sugammadex and acetylcholinesterase inhibitors. Illman et al. [29] reported that the time from reversal agent administration during T2 detection via TOF (train of four) monitoring to the time of residual paralysis recovery was 13.3 min for neostigmine and 1.7 min for sugammadex [29]. Assuming an average PACU stay time of 30 min, residual paralysis following PACU discharge may have little effect on respiratory clinical outcomes. In summary, sugammadex may not offer advantages over acetylcholinesterase inhibitors in terms of respiratory complications, though further prospective clinical trials assessing postoperative pulmonary function after sugammadex use remain necessary.

The incidence of delayed discharge was lower in the sugammadex group in the present study; the precise factors associated with delayed discharge were different between the two groups. The leading cause for delayed discharge in the acetylcholinesterase group was ileus (90%), while in the sugammadex group, ileus accounted for far fewer incidences of delayed discharge (39.1%). Postoperative ileus following colorectal surgery is a common complication that presents in one in every eight patients and is a primary contributor to delayed discharge [30].

The exact factor which resulted in group-wise differences between the ileus rates in the present study is likely multifactorial and complex. The retrospective nature of the present study’s design, however, does not allow for determining this factor, though a possibility is the effect of reversal agents on gastrointestinal motility. Acetylcholinesterase inhibitors and anticholinergics are commonly used to reverse neuromuscular blockade. Both drugs have a known impact on gastrointestinal motility—where acetylcholinesterase inhibitors increase motility, anticholinergic agents decrease it [31,32]. This leads to variable effects on gastrointestinal motility during the perioperative period.

While sugammadex is known to have no effects on gastric emptying, one study found that it binds to progesterone and estradiol. The former enhances gastric motility while the latter inhibits it. Sugammadex also traps more estrogen than progesterone, leading to increase gastric emptying. Several studies regarding gastrointestinal motility with sugammadex and anticholinesterase inhibitor use in the postoperative period have been published. Brueckmann et al. reported similar incidences of ileus (3.9–4.1%) and paralytic ileus (0–1.3%) in patients following abdominal surgery [9]. Sen et al. [33] reported that there was no difference in first flatus time and feces time in the postoperative period in thyroidectomy patients. In one report, the authors reported a tendency towards faster gastric emptying in sugammadex users than in neostigmine/atropine users [34]. In addition, early feeding and opioid avoidance strategies might affect postoperative ileus [35]. While we did not consider opioid use here, further studies of sugammadex’s effects on delayed postoperative ileus and delayed discharge following abdominal surgery are required.

Limitations of this study are as follows. First of all, the present study’s use of a retrospective design is a significant limitation, as is the time between when data was collected on patients in the two groups. Secondly, the present study’s relatively small sample size is also a limitation. All surgical procedures for this present study, however, were carried out by a single surgeon with over 30 years of experience, and postoperative protocols did not differ among the two groups, with the exception of reversal agent type. Thirdly, the term “delayed discharge” used in our text (i.e., >11 days of hospitalization) is an arbitrary term. Total hospitalization times for colorectal patients differ between studies. We have defined “delayed discharge” as more than mean value, since our center’s postoperative admission protocol, as well as mean postoperative hospitalization time, is 10 days. Fourthly, the exact dose of rocuronium and pyridostigmine was also not recorded, while doses of sugammadex were constantly 200 mg. Lastly, neuromuscular monitoring may have provided further information, and future studies might seek to integrate this outcome. A well-designed future prospective study is required to further determine the long-term effects of sugammadex.

## 5. Conclusions

In conclusion, the present study aimed to compare the 30-day postoperative outcomes of surgical patients treated with either sugammadex or acetylcholinesterase inhibitor use. We found no differences in total length of hospital stay, length of postoperative hospital stay, readmission rates, or postoperative pulmonary complication rates between the two groups. In fact, the only difference between the two groups was the incidence of delayed discharge, which was lower in the sugammadex group. These results should be considered when the long-term benefits of sugammadex are discussed.

## Figures and Tables

**Table 1:** Baseline characteristics.

Total (*n* = 585)	Before Matching			After Matching		
	Group S (*n* = 201)	Group A (*n* = 384)	*p* Value	Group S (*n* = 157)	Group A (*n* = 157)	*p* Value
Age (years)	63.7 ± 11.3	61.5 ± 12.1	0.037	62.5 ± 11.5	63.1 ± 11.8	0.67
Sex, male	110 (55)	210 (55)	0.99	86 (55)	83 (53)	0.73
Body mass index (kg/m^2^)	23.8 ± 3.4	23.5 ± 3.2	0.24	23.8 ± 3.3	23.4 ± 3.4	0.27
ASA			0.085			0.26
1	87 (43)	195 (51)		67 (43)	77 (49)	
2	114 (57)	189 (49)		90 (57)	80 (51)	
Comorbidities						
Cardiovascular	96 (48)	162 (42)	0.20	76 (48)	70 (45)	0.50
Diabetes	40 (20)	61 (16)	0.22	30 (19)	26 (17)	0.56
Cerebrovascular	7 (3)	13 (3)	0.95	6 (4)	3 (2)	0.31
Pulmonary	10 (5)	9 (2)	0.088	6 (4)	2 (1)	0.15
Operation type			0.012			0.78
Laparoscopic	39 (19)	45 (12)		32 (20)	34 (22)	
Open	162 (81)	339 (88)		125 (80)	123 (78)	
Anesthesia time (min)	181.0 ± 48.6	165.1 ± 41.7	<0.001	176.0 ± 46.7	175.1 ± 41.0	0.85

Values are mean ± standard deviation or number (%). Group S, sugammadex group; Group A, control group using acetylcholinesterase inhibitor; ASA, American Society of Anesthesiologists physical status.

**Table 2:** Postoperative outcomes.

Total (*n* = 585)	Before Matching			After Matching		
	Group S (*n* = 201)	Group A (*n* = 384)	*p* Value	Group S (*n* = 157)	Group A (*n* = 157)	*p* Value
Primary outcomes						
Total length of stay (day)	13.3 ± 12.3	13.3 ± 5.0	0.95	13.2 ± 12.9	13.4 ± 4.2	0.84
Postoperative hospital stay (day)	10.3 ± 12.2	9.9 ± 4.9	0.59	10.1 ± 12.9	10.0 ± 4.0	0.88
Delayed discharge, *n* (%)	32 (16)	90 (23)	0.034	23 (15)	40 (25)	0.017
Readmission rate, *n* (%)	18 (9)	29 (8)	0.55	12 (8)	15 (10)	0.55
Secondary outcomes						
PACU stay time (min)	46.9 ± 21.0	49.3 ± 20.5	0.172	50.0 ± 24.5	49.3 ± 20.5	0.821
ICU admission rate, *n* (%)	7 (3)	4 (1)	0.044	4 (3)	2 (1)	0.41
Mortality, *n* (%)	2 (1)	2 (1)	0.51	2 (1)	1 (1)	0.56
Oral intake						
Sips of water (hour)	94.9 ± 82.6	96.8 ± 67.9	0.76	87.9 ± 58.3	98.5 ± 79.5	0.18
Meal (hour)	122.5 ± 84.8	123.5 ± 68.3	0.88	115.8 ± 58.3	126.7 ± 78.7	0.16
Complications						
Pulmonary	6 (3)	1 (0)	0.004	4 (3)	1 (1)	0.18

Values are mean ± standard deviation or number (%). Group S, sugammadex group; Group A, control group using acetylcholinesterase inhibitor; PACU, postanesthetic care unit; ICU, intensive care unit.

**Table 3:** Causes of delayed discharge.

Causes	Total Cases (*n* = 63)	Group S (*n* = 23)	Group A (*n* = 40)
Ileus	45 (71.4)	9 (39.1)	36 (90.0)
Pneumonia	5 (7.9)	4 (17.4)	1 (2.5)
Wound problem	6 (9.5)	4 (17.4)	2 (5.0)
Urinary tract infection	2 (3.2)	2 (8.7)	0 (2.2)
Postoperative bleeding	2 (3.2)	2 (8.7)	0 (0)
Urinary retention	2 (3.2)	2 (8.7)	0 (0)
Pain	1 (1.6)	0 (0)	1 (2.5)

Values are mean ± standard deviation or number (%). Group S, sugammadex group; Group A, control group using acetylcholinesterase inhibitor.

## References

[1] Murphy G.S., Szokol J.W., Marymont J.H., Greenberg S.B., Avram M.J., Vender J.S. (2008). Residual neuromuscular blockade and critical respiratory events in the postanesthesia care unit. Anesth. Analg..

[2] Butterly A., Bittner E.A., George E., Sandberg W.S., Eikermann M., Schmidt U. (2010). Postoperative residual curarization from intermediate-acting neuromuscular blocking agents delays recovery room discharge. Br. J. Anaesth..

[3] Hayes A.H., Mirakhur R.K., Breslin D.S., Reid J.E., McCourt K.C. (2001). Postoperative residual block after intermediate-acting neuromuscular blocking drugs. Anaesthesia.

[4] Fortier L.P., McKeen D., Turner K., de Medicis E., Warriner B., Jones P.M., Chaput A., Pouliot J.F., Galarneau A. (2015). The RECITE Study: A Canadian Prospective, Multicenter Study of the Incidence and Severity of Residual Neuromuscular Blockade. Anesth. Analg..

[5] Varposhti M.R., Heidari S.M., Safavi M., Honarmand A., Raeesi S. (2011). Postoperative residual block in postanesthesia care unit more than two hours after the administration of a single intubating dose of atracurium. J. Res. Med. Sci..

[6] Madsen M.V., Scheppan S., Mork E., Kissmeyer P., Rosenberg J., Gatke M.R. (2017). Influence of deep neuromuscular block on the surgeons assessment of surgical conditions during laparotomy: A randomized controlled double blinded trial with rocuronium and sugammadex. Br. J. Anaesth..

[7] King M., Sujirattanawimol N., Danielson D.R., Hall B.A., Schroeder D.R., Warner D.O. (2000). Requirements for muscle relaxants during radical retropubic prostatectomy. Anesthesiology.

[8] Putz L., Dransart C., Jamart J., Marotta M.L., Delnooz G., Dubois P.E. (2016). Operating room discharge after deep neuromuscular block reversed with sugammadex compared with shallow block reversed with neostigmine: A randomized controlled trial. J. Clin. Anesth..

[9] Brueckmann B., Sasaki N., Grobara P., Li M.K., Woo T., de Bie J., Maktabi M., Lee J., Kwo J., Pino R. (2015). Effects of sugammadex on incidence of postoperative residual neuromuscular blockade: A randomized, controlled study. Br. J. Anaesth..

[10] Carron M., Zarantonello F., Lazzarotto N., Tellaroli P., Ori C. (2017). Role of sugammadex in accelerating postoperative discharge: A meta-analysis. J. Clin. Anesth..

[11] Carron M., Veronese S., Foletto M., Ori C. (2013). Sugammadex allows fast-track bariatric surgery. Obes. Surg..

[12] Unal D.Y., Baran I., Mutlu M., Ural G., Akkaya T., Ozlu O. (2015). Comparison of sugammadex versus neostigmine costs and respiratory complications in patients with obstructive sleep apnoea. Turk. J. Anaesthesiol. Reanim..

[13] Thevathasan T., Shih S.L., Safavi K.C., Berger D.L., Burns S.M., Grabitz S.D., Glidden R.S., Zafonte R.D., Eikermann M., Schneider J.C. (2017). Association between intraoperative non-depolarising neuromuscular blocking agent dose and 30-day readmission after abdominal surgery. Br. J. Anaesth..

[14] Kotake Y., Ochiai R., Suzuki T., Ogawa S., Takagi S., Ozaki M., Nakatsuka I., Takeda J. (2013). Reversal with sugammadex in the absence of monitoring did not preclude residual neuromuscular block. Anesth. Analg..

[15] Martinez-Ubieto J., Ortega-Lucea S., Pascual-Bellosta A., Arazo-Iglesias I., Gil-Bona J., Jimenez-Bernardo T., Munoz-Rodriguez L. (2016). Prospective study of residual neuromuscular block and postoperative respiratory complications in patients reversed with neostigmine versus sugammadex. Minerva Anestesiol..

[16] Della Rocca G., Pompei L., Pagan D.E.P.C., Tesoro S., Mendola C., Boninsegni P., Tempia A., Manstretta S., Zamidei L., Gratarola A. (2013). Reversal of rocuronium induced neuromuscular block with sugammadex or neostigmine: A large observational study. Acta Anaesthesiol. Scand..

[17] O’Reilly-Shah V.N., Lynde G.C., Mitchell M.L., Maffeo C.L., Jabaley C.S., Wolf F.A. (2018). Initial experience with the unrestricted introduction of sugammadex at a large academic medical center: A retrospective observational study examining postoperative mechanical ventilation and efficiency outcomes. Korean J. Anesthesiol..

[18] Ledowski T., Hillyard S., O’Dea B., Archer R., Vilas-Boas F., Kyle B. (2013). Introduction of sugammadex as standard reversal agent: Impact on the incidence of residual neuromuscular blockade and postoperative patient outcome. Indian J. Anaesth..

[19] Geldner G., Niskanen M., Laurila P., Mizikov V., Hubler M., Beck G., Rietbergen H., Nicolayenko E. (2012). A randomised controlled trial comparing sugammadex and neostigmine at different depths of neuromuscular blockade in patients undergoing laparoscopic surgery. Anaesthesia.

[20] Jones R.K., Caldwell J.E., Brull S.J., Soto R.G. (2008). Reversal of profound rocuronium-induced blockade with sugammadex: A randomized comparison with neostigmine. Anesthesiology.

[21] Hristovska A.M., Duch P., Allingstrup M., Afshari A. (2017). Efficacy and safety of sugammadex versus neostigmine in reversing neuromuscular blockade in adults. Cochrane Database Syst. Rev..

[22] Blobner M., Eriksson L.I., Scholz J., Motsch J., Della Rocca G., Prins M.E. (2010). Reversal of rocuronium-induced neuromuscular blockade with sugammadex compared with neostigmine during sevoflurane anaesthesia: Results of a randomised, controlled trial. Eur. J. Anaesthesiol..

[23] Watts R.W., London J.A., van Wijk R.M., Lui Y.L. (2012). The influence of unrestricted use of sugammadex on clinical anaesthetic practice in a tertiary teaching hospital. Anaesth. Intensive Care.

[24] Ledowski T., Falke L., Johnston F., Gillies E., Greenaway M., De Mel A., Tiong W.S., Phillips M. (2014). Retrospective investigation of postoperative outcome after reversal of residual neuromuscular blockade: Sugammadex, neostigmine or no reversal. Eur. J. Anaesthesiol..

[25] Waddle J.P., Evers A.S., Piccirillo J.F. (1998). Postanesthesia care unit length of stay: Quantifying and assessing dependent factors. Anesth. Analg..

[26] Berg H., Roed J., Viby-Mogensen J., Mortensen C.R., Engbaek J., Skovgaard L.T., Krintel J.J. (1997). Residual neuromuscular block is a risk factor for postoperative pulmonary complications. A prospective, randomised, and blinded study of postoperative pulmonary complications after atracurium, vecuronium and pancuronium. Acta Anaesthesiol. Scand..

[27] Kirmeier E., Eriksson L.I., Lewald H., Jonsson Fagerlund M., Hoeft A., Hollmann M., Meistelman C., Hunter J.M., Ulm K., Blobner M. (2018). Post-anaesthesia pulmonary complications after use of muscle relaxants (POPULAR): A multicentre, prospective observational study. Lancet Respir. Med..

[28] Abad-Gurumeta A. (2018). Evidence of residual neuromuscular block with sugammadex vs neostigmine. Br. J. Anaesth..

[29] Illman H.L., Laurila P., Antila H., Meretoja O.A., Alahuhta S., Olkkola K.T. (2011). The duration of residual neuromuscular block after administration of neostigmine or sugammadex at two visible twitches during train-of-four monitoring. Anesth. Analg..

[30] Scarborough J.E., Schumacher J., Kent K.C., Heise C.P., Greenberg C.C. (2017). Associations of Specific Postoperative Complications With Outcomes After Elective Colon Resection: A Procedure-Targeted Approach Toward Surgical Quality Improvement. JAMA Surg..

[31] Manini M.L., Camilleri M., Grothe R., Di Lorenzo C. (2018). Application of pyridostigmine in pediatric gastrointestinal motility disorders: A case series. Paediatr. Drugs.

[32] Donnellan C.M.B. (2006). Effect of Atropine and Glycopyrrolate in Ameliorating the Clinical Signs Associated with the Inhibition of Cholinesterase Activity by Imidocarb Dipropionate in Horses. Master’s Thesis.

[33] Sen A., Erdivanli B., Tomak Y., Pergel A. (2016). Reversal of neuromuscular blockade with sugammadex or neostigmine/atropine: Effect on postoperative gastrointestinal motility. J. Clin. Anesth..

[34] Sustic A., Dijana D. (2012). Early postoperative gastric emptying in patients undergoing laparoscopic cholecystectomy: Sugammadex vs. neostigmine/atropine neuromuscular blockade reversal agents: 9AP4-1. Eur. J. Anaesthesiol..

[35] Chapman S.J., Pericleous A., Downey C., Jayne D.G. (2018). Postoperative ileus following major colorectal surgery. Br. J. Surg..

